# Chk1/2 Inhibition by AZD7762 Enhances Andrographolide-Induced DNA Damage, Impairs DNA Repair, and Promotes Apoptosis in Head and Neck Squamous Cell Carcinoma CAL-33 Cells

**DOI:** 10.3390/cancers18193199

**Published:** 2026-10-03

**Authors:** Sakshi Singh, Anitha Kartha, Aishwarya Jaiswal, Dhanir Tailor, Sivanandhan Dhanalakshmi

**Affiliations:** 1 School of Biotechnology, Gautam Buddha University, Greater Noida 201312, UP, India; 241dbt005@gbu.ac.in; 2 Cancer Biology Laboratory, Department of Life Sciences, Central University of Gujarat, Gandhinagar 382030, GJ, India; karthaanitha@gmail.com; 3 Cancer Biology Laboratory, School of Life Sciences, Jawaharlal Nehru University, New Delhi 110067, India; aishwa19_sls@jnu.ac.in; 4 Center for Experimental Therapeutics, Oregon Health and Science University, Portland, OR 97239, USA; tailor@ohsu.edu

**Keywords:** HNSCC, DNA damage, andrographolide, Chk1/2 inhibition, apoptosis

## Abstract

**Background**: Andrographolide (AG), a diterpenoid lactone and a major constituent of *Andrographis paniculata*, has shown promising antiproliferative effect on diverse cancer cell lines, including head and neck cancer. However, a significant anti-proliferative effect is observed only at high micromolar concentrations. Further, AG has been shown to have poor oral bioavailability. Therefore, it is essential to consider mechanism-based combinations that could synergistically enhance the therapeutic potential of this compound so that efficacy can be achieved in concentrations that are systemically achievable. **Objective**: The present study is aimed to evaluate the anticancer effects and associated molecular alterations of an AG and Chk1/2 inhibitor combination in head and neck squamous cell carcinoma (CAL-33) cells since the earlier studies have suggested ATM/ATR/Chk1/2-mediated activation of DNA damage checkpoints. **Methods**: Normal FHs74int cells and HNSCC cells (CAL-33 and FaDu) were used to assess cell proliferation and cell death. Cell cycle distribution and apoptosis were analyzed by flow cytometry and Acridine orange/EtBr, respectively, in CAL-33 cells. DNA damage in CAL-33 and FaDu cells was assessed by comet assay. Changes in the expression of proteins implicated in apoptosis, cell cycle progression, and DNA damage signaling were examined using Western blotting. **Results**: While AG did not show any significant changes in the growth or cell death of normal FHs74int cells, it strongly inhibited proliferation of HNSCC cells, concomitant with G2/M arrest and apoptosis. AG and AZD7762 showed an IC50 of 2.743 μM and 0.02600 μM, respectively, on cell proliferation in CAL-33 cells. Furthermore, AG caused an accumulation of intracellular ROS, which was inhibited by treatment with N-acetylcysteine. Chk1, Chk2 and γH2AX phosphorylation were induced in a dose-dependent manner by treatment with AG. The combination of AG with Chk1/2 inhibitor AZD7762 showed a synergistic effect in inhibiting cell proliferation with a CI of 0.4 through inhibition of DNA repair and premature mitotic entry, ultimately resulting in programmed cell death. **Conclusions**: These findings provide evidence that AG selectively targets DNA damage and repair in HNSCC CAL-33 cells via cell cycle arrest and apoptosis. Further, the anticancer potential of AG can be significantly enhanced by combination with the Chk1/2 inhibitor AZD7762, preventing repair of AG-induced DNA damage and amplifying checkpoint stress responses, resulting in enhanced apoptotic cell death. Together, these findings support the therapeutic potential of AG and highlight checkpoint kinase inhibition as a rational combination strategy for HNSCC treatment.

## Simple Summary

Over 90% of all head and neck cancers are head and neck squamous cell carcinoma (HNSCC), the sixth most frequent cancer worldwide. HNSCC is associated with poor prognosis due to therapeutic resistance, tumor recurrence, and efficient DNA damage repair mechanisms that limit the efficacy of conventional treatments. Although andrographolide exhibits anticancer activity by triggering apoptosis and G2/M cell cycle arrest, its therapeutic potential is restricted by its low bioavailability and activation of the Chk1/Chk2 checkpoint pathway and DNA damage response. In our study we used the Chk1/2 inhibitor AZD7762 in combination with andrographolide as a promising strategy that resulted in increased DNA damage and apoptosis thereby leading to enhanced anticancer efficacy, even at lower drug concentrations, in CAL-33 cells.

## 1. Introduction

Chemoprevention of cancer focuses on cancer control through pharmacological and nutritional interventions involving natural or synthetic agents. Worldwide, head and neck squamous cell carcinoma (HNSCC) is the sixth most common cancer with 300,000 deaths each year [1]. In India, it is prevalent and ranks as the second most common cancer among males [2]. The incidence rates of HNSCC are found to be highest in regions with increased rates of alcohol and tobacco consumption [3]. Even though patients have benefited largely from the advances in the treatment modalities, the 5-year survival rate remains at 50% since the past few decades, despite surgery, chemotherapy, and radiation therapy [4]. Acute and long-term complications associated with existing cancer treatment modalities necessitate the search for an alternative approach that is affordable, effective and less toxic. Cancer of the hypopharynx has worst prognosis among head and neck cancers [5]. Malignancies of the oral cavity and pharynx account for more than two-thirds of head and neck cancer cases and fatalities [6].

Numerous epidemiological studies have indicated that the intake of vegetables, fruits and phytochemicals from non-dietary sources provides protection against different types of cancer [7,8,9]. In this context, *Andrographis paniculata* Nees, a widely recognized plant from the Indian traditional system of medicines, has shown preventive potential against chemotoxicity including carcinogenicity [10]. Andrographolide (AG), a major diterpenoid constituent of *A. paniculata*, has shown anti-inflammatory and anticancer activities in varieties of human cancer cell lines including epidermoid carcinoma KB cells, lymphocytic leukemia P388 cells, colon carcinoma HCT116 and COLO205 cells [11,12]. Recent studies indicate its efficacy against HNSCC [13]. However, its effects and associated mechanisms are not completely understood in head and neck cancer.

Although andrographolide’s (AG) anticancer properties have been thoroughly studied, its pharmacokinetic fate and biological variables impact its bioavailability. The absolute oral bioavailability of AG is poor. Rapid first-pass metabolism to a sulfate metabolite in the liver and gut is the main cause of its poor bioavailability [14]. Therefore, novel approaches are required to increase its potency and efficacy. Several studies have shown that modulation of the cell cycle checkpoint and the associated DNA damage response negatively impact the anticancer activity of chemotherapeutic and chemopreventive agents. Our earlier studies have shown that AG treatment induced G2M cell cycle arrest and since Chk1/2 proteins play a critical role in G2M arrest, we planned to evaluate a Chk1/2 inhibitor in combination with AG. AZD7762 is a potent, ATP-competitive Chk1/Chk2 inhibitor evaluated to enhance cancer cell death. It is primarily studied in combination with DNA-damaging agents like gemcitabine [15], platinum chemotherapies like neoadjuvant carboplatin in triple-negative breast cancer [16], and radiation therapy [17] to abrogate the cell cycle’s G2/M arrest, impair DNA repair, and trigger apoptosis.

Here, we report for the first time the evaluation of andrographolide (AG) in combination with the Chk1/2 inhibitor AZD7762 in CAL-33 cells to determine its effect on cell viability, cell cycle distribution, DNA damage and apoptosis-associated mechanisms.

## 2. Materials and Methods

### 2.1. Cell Culture and Treatments

CAL-33 (tongue squamous cell carcinoma), FaDu cells (human hypopharyngeal cells) and FHs74int cells (non-neoplastic intestinal cells) were procured from American Type Culture Collection (Manassas, VA, USA). CAL-33 and FaDu cells were grown in DMEM media, and FHs74int cells were maintained in hybrid-care media containing recommended concentrations of growth factors (10 ng/mL EGF) [18]. At 70% confluency, cultures were treated with AG dissolved in DMSO to prepare stock solution. Working concentrations of 5, 10, and 25 µM AG (Sigma Aldrich, St. Louis, MO, USA) were freshly prepared through diluting the stock in complete DMEM culture media for desired time points (24–72 h) for cell proliferation and 24 h for cell cycle analysis. CAL-33 was treated with 10 decreasing concentrations at 3-fold dilution with a starting concentration of 100 μM for AG and 30 μM for AZD7762 for dose–response curve study for MTT assay. Cells were treated with an equivalent concentration of DMSO, which served as the vehicle control. Doxorubicin was used as a positive control for MTT cell viability assay. Where mentioned, cells were treated with 10 mM NAC (Sigma Aldrich) 3 h prior to AG treatment. CAL-33 were treated with AZD7762 (Cayman Chemicals, Ann Arbor, MI, USA) inhibitor in combination with andrographolide at much lower doses (1AG, 10AG, 0.1Chk, 0.3Chk) in µM.

### 2.2. Cell Growth and Death Assays

CAL-33 and FaDu cells (10^5^ cells in 65 mm culture plate) were seeded in complete DMEM medium and after 24 h treated with 5–25 μM AG for 24–72 h and 24–48 h, respectively, for cell growth and cell death assays. Similarly FHs74int cells (10^5^/65 mm culture plate) were seeded in DMEM supplemented with 10% Fetal Bovine Serum (FBS), 10 μg/mL insulin and 30 ng/mL EGF, and after 24 h were treated with 5–25 μM AG for 24–48 h. Cells were collected, processed and counted after trypan blue staining as published earlier [19].

### 2.3. MTT Assay

To evaluate the cell proliferation IC50s of AG and AZD7762, CAL-33 were plated in 96-well plates at a density of 5000 cells/well for 24 h, treated with 10 decreasing concentrations at 3-fold dilution with a starting concentration of 100 μM for AG and 30 μM for AZD7762. Proliferation studies to assess combination effects of AG and AZD7762 were carried out at in a similar manner with 1, 0.33, 0.11, 0.033 and 0.011 μM of AZD7762, and 10, 3.3, 1.1, 0.33 and 0.11 μM of AG, respectively, treated alone and in combination. MTT [3-(4,5-dimethylthiazol-2-yl)-2,5-diphenyltetrazolium bromide; Sigma] assay was performed in accordance with the manufacturer’s guidelines. Briefly, after 72 h of treatment, 50 μL of 5 mg/mL MTT reagent was added and incubated for 3–4 h. The MTT reagent was removed and 100 μL of 99.98% DMSO was added to each well and incubated at room temperature for 10 min. Absorbance was measured at 575 nm by using a BioTek microplate reader (BioTek, Winooski, VT, USA) [20].

### 2.4. Apoptotic Cell Death Assay

FaDu cells were treated with AG (5–25 µM) for 48 h and then harvested. Apoptosis was determined quantitatively using the Annexin V-FITC Apoptosis Detection Kit by flow cytometry following manufacturer’s protocol as previously described [21].

### 2.5. Assessment of Mitochondrial Membrane Potential Using JC-1 Staining

Briefly, 70% confluent FaDu cells were treated with 5–25 µM AG for 24 h and harvested. Cells (1 × 10^5^) were suspended in 1 mL PBS containing lipophilic cationic probe JC-1 dye and incubated in the dark at 37 °C for 15 min. The fluorescence of JC-1 monomers and aggregates was measured by flow cytometry, and the red/green fluorescence intensity ratio was calculated to assess mitochondrial membrane depolarization according to the manufacturer’s instructions and previously described methods [21].

### 2.6. Immunoblotting

CAL-33 and FaDu cells were treated at 70% confluency with mentioned doses of AG and/or NAC, H_2_O_2_ and AZD7762 for 48 h as indicated in the figures. At the end of the treatment, cell lysate was prepared using lysis buffer and Western immunoblotting was performed as described earlier [22].

### 2.7. COMET Assay

After desired treatment, CAL-33 and FaDu cells were gently mixed in 1% low melting point agarose and coated onto a microscope slide. The microscopic slides were then dipped in freshly prepared lysis buffer for an hour, after which the slides were dipped for 20 min in unwinding buffer at 4 °C. Thereafter, the slides were immersed for 20 min in neutralization buffer and were run on horizontal gel electrophoresis, stained with 1 mg/mL ethidium bromide [23], dried and observed under fluorescence microscope. Images were captured and the extent of DNA damage was measured by scoring the comet tails using CometScore 2.0 application [22].

### 2.8. Acridine Orange (AO)/EtBr Apoptosis Staining

CAL-33 cells were treated with AG (10,25 µM) for 48 h. Apoptosis was determined quantitatively using the AO/EtBr dye. Cells were collected by brief trypsinization after 48 h of treatment, centrifuged at 1500 rpm, and the pellet collected was resuspended in 50 μL of PBS. Next, 25 μL of the cell suspension and 2 μL of AO/EtBr solution were mixed gently. Immediately, 20 μL of the mixed stained solution was pipetted onto a glass slide and evenly spread using a coverslip. The slides were examined under a Olympus fluorescence microscope and cells were counted. The population of apoptotic cells was determined and expressed as a percentage of the total cells counted, as described previously.

### 2.9. Cell Cycle Phase Distribution Analysis by FACS

CAL-33 cells were seeded and treated with AZD7762 (0.1 and 0.3 μM) and AG (1 and 10 μM) either alone or in combination (AG 1 μM + 0. 1 or 0.3 μM AZD7762 and AG 10 μM + 0.1 or 0.3 μM AZD7762) for 24 h. Following treatment, cells were harvested, centrifuged at 1500 rpm, and cell pellet was processed for cell cycle analysis as described earlier [19]. Briefly, after washing with 1x PBS, cells were centrifuged and resuspended in 500 µL of saponin–PI solution as reported earlier [24], and incubated in dark overnight at 4 °C. Cell cycle analysis of these cells was performed using BD FACS Lyric flow cytometer (BD Biosciences, San Jose, CA, USA).

### 2.10. Data Analysis

GraphPad Prism version 8 was used to conduct statistical analysis. Student’s *t*-test was used to compare the control groups and treatment groups. The data obtained from MTT was log-transformed and four-parameter sigmoidal dose–response curve (variable slope model) parameter was applied using GraphPad Prism version 8. MTT assay robustness was ensured by maintaining a SW of >2 and Z′ factor of >0.4. A statistically significant difference was considered as *p* < 0.05. All experiments were repeated 2–3 times to ensure the reproducibility of results.

## 3. Results

### 3.1. AG Inhibits Growth and Increases Cell Death Selectively in CAL-33 and FaDu Cells

**Figure 1:**
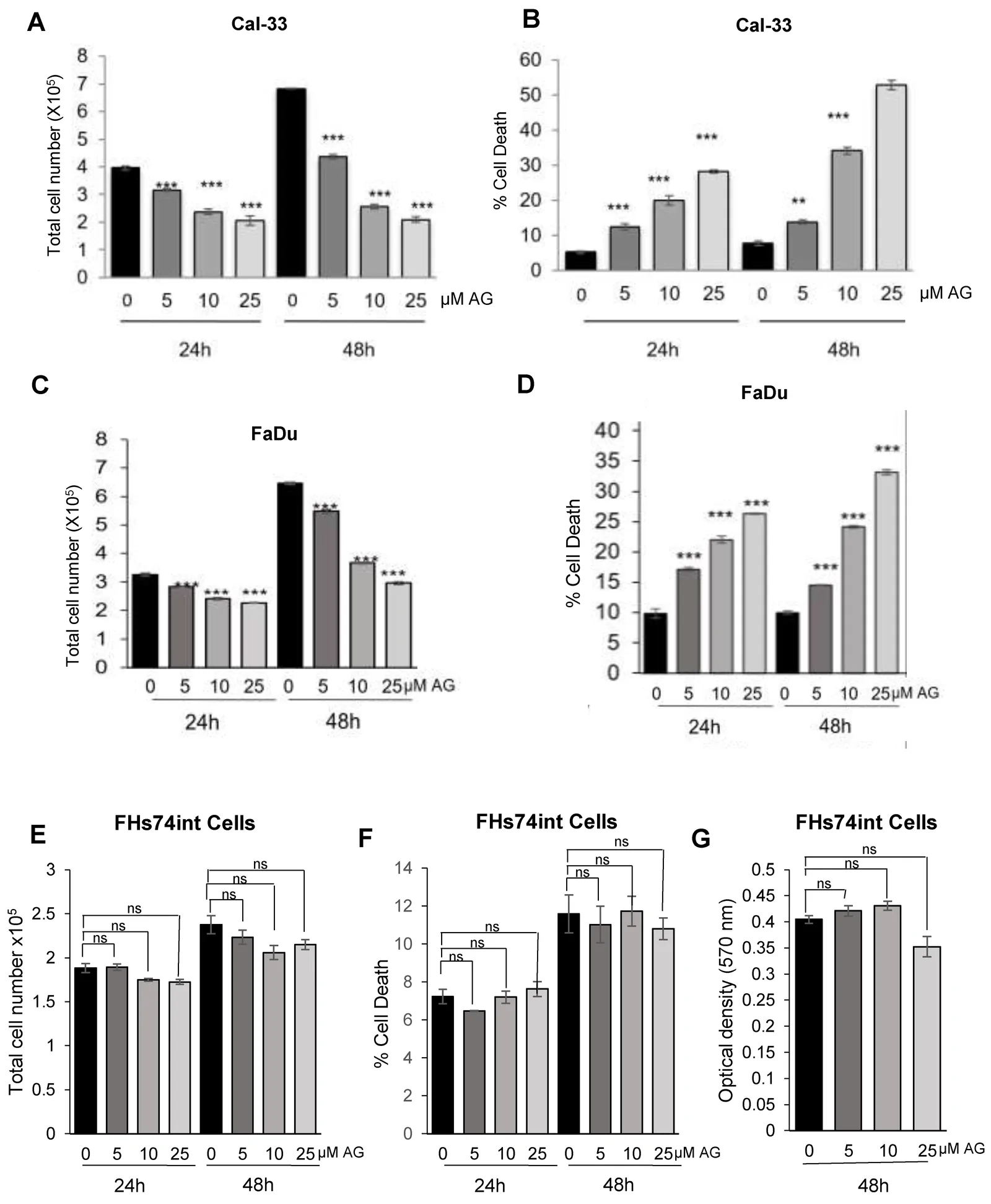
Effect of andrographolide (AG) on growth and death of CAL-33, FaDu cells, and normal human intestinal epithelial FHs74int cells. For 24 to 72 h, cells were treated with DMSO (control), 5, 10, and 25 μM AG. Using a hemocytometer, total cells were counted and dead cells were calculated using the trypan blue dye exclusion method. (**A**) Total cell number and (**B**) percentage cell death at 24 and 48 h of AG treatments in CAL-33 cells. (**C**) Total cell number and (**D**) percentage cell death at 24, and 48 of AG treatments of FaDu cells. (**E**) Total cell number and (**F**) percentage cell death at 24, 48 h of AG treatments of FHs74int cells. (**G**) Cell viability of FHs74int cells by MTT assay. Results are representative of three independent experiments. For each treatment, the data are displayed as means of triplicate samples. Bars, SE; ** *p* < 0.01, *** *p* < 0.001 when compared to the control.

AG treatment of CAL-33 cells decreased the total number of cells and increased cell death in a time- and dose-dependent manner (Figure 1A,B). AG (5–25 μM) treatment for 24 h and 48 h decreased the total cell number by 21–47% (*p* < 0.001) and 36–69% (*p* < 0.001; Figure S1), respectively. AG treatment increased cell death in CAL-33 cells by 7–23% and 6–36% (*p* < 0.01–0.001) at 24 h and 48 h, respectively (Figure 1B).

AG treatment of FaDu cells decreased the total number of cells and increased cell death in a time- and concentration-dependent manner (Figure 1C,D). AG (5–25 μM) treatment of FaDu cells reduced the total number of cells by 14–30% (*p* < 0.001), and 16–54% (*p* < 0.001) (Figure 1C) and cell death was increased by 7–16% and 4–23% at 24 and 48 h, respectively (Figure 1D).

AG treatment of human non-cancerous FHs74int cells at the same concentrations and time durations did not exhibit considerable effects on the total cell number or cell death (Figure 1E,F), which was confirmed by the MTT assay (Figure 1G). These results suggest that AG selectively inhibits growth and induces death in CAL-33 and FaDu cells.

### 3.2. Effect of AG on Cell Cycle Progression and Apoptotic Death of FaDu Cells

**Figure 2:**
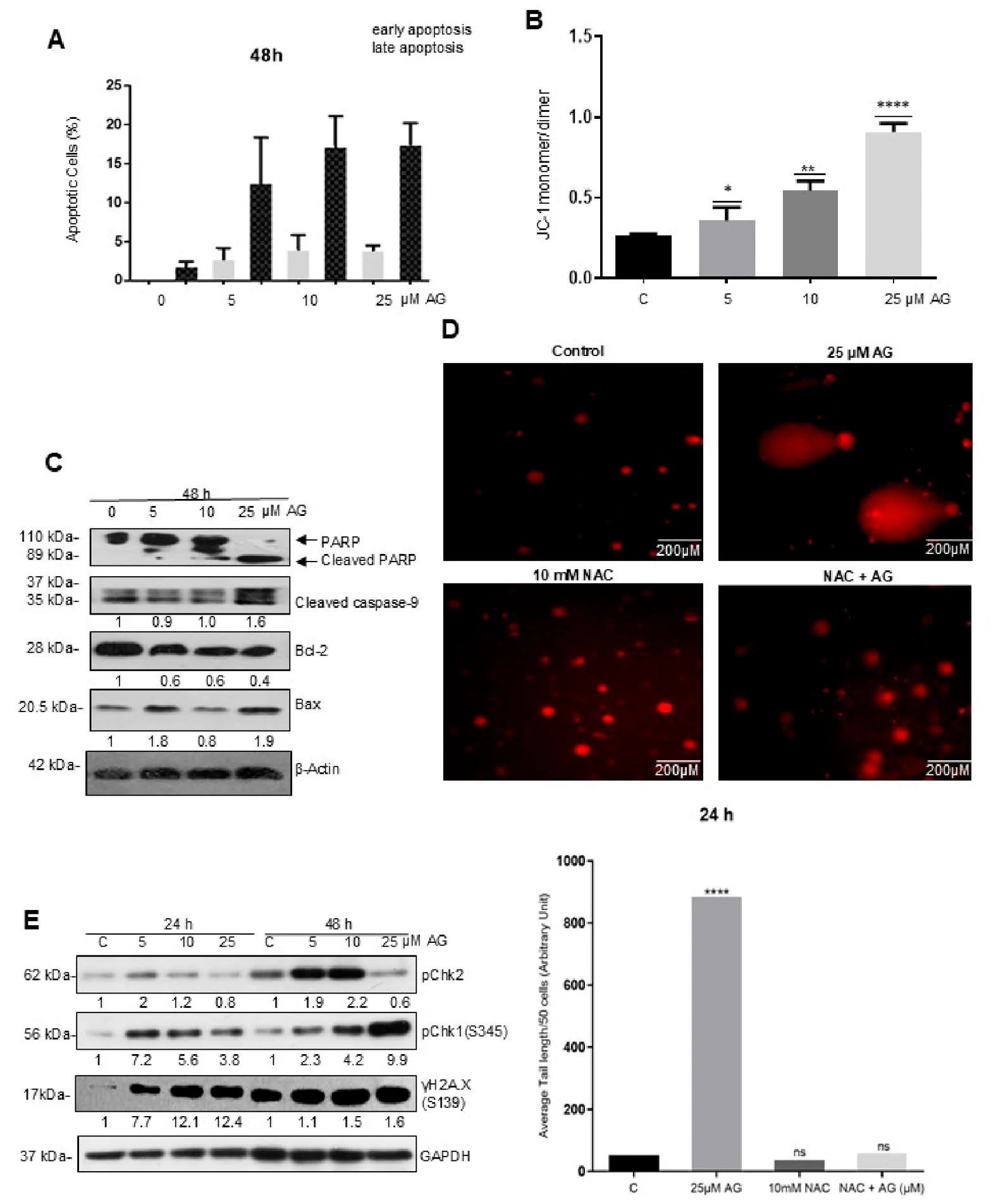
Effect of AG on cell cycle progression and apoptotic death of FaDu cells. The cells were treated with 5, 10 or 25 μM AG for 24–72 h. Following treatment, cells were collected and stained with PI and annexin V. Percentage of apoptotic cells was determined by flow cytometry. (**A**) The mean of triplicate samples representing the percentage of apoptotic cells (annexin V-positive). (**B**) JC-1 monomer/dimer ratio are expressed as mean of 590 nm/520 nm emission spectrum of triplicate samples. (**C**) Western immunoblot analysis of Bax, Bcl-2, cleaved-poly (ADP-ribose) polymerase (PARP), total PARP, and cleaved caspase 9, with β-actin used as loading control. The densitometric data shown below each band are fold-change in the protein level normalized to β-actin. Effect of AG on mitochondrial membrane potential in FaDu cells and on checkpoint signaling. Cells were treated with 5, 10 or 25 μM AG for 48 h. (**D**) Comet assay was done in FaDu cells at 24 h of AG treatment (25 µM), photographs were captured at 400× magnification as described in Materials and Methods, and tail length measured; scale, 200 µM. The histogram below represents the average tail length of 50 cells per group, expressed in arbitrary units (AUs). (**E**) Western immunoblot of pChk2, pChk1, phospho-H2A.X(S139) and GAPDH as control. Bars, SE; *n* = 3, * *p* < 0.05, ** *p* < 0.01 and **** *p* < 0.0001 versus control.

Consistent with the cell death, AG (5–25 μM) treatment at 48 h increased apoptotic cells to 14–21% (*p* < 0.05–0.01) as compared to 2% in the control (Figure 2A). The loss of the mitochondrial membrane potential marks the initiation and activation of mitochondria-mediated apoptosis [25]. AG (5–25 μM) treatment for 24 h resulted in dissociation of JC-1 from its dimeric to monomeric form (Figure 2B), suggesting a clear loss in mitochondrial membrane integrity [24]. The monomer/dimer ratio of JC-1 increased significantly up to four-fold (*p* < 0.01–0.001) (Figure 2B), which confirmed earlier observations of apoptosis induction by AG in FaDu cells. Accordingly, AG increased the levels of cleaved caspase-9 and cleaved PARP (Figure 2C). This finding suggested that AG-induced apoptosis involved both caspase-dependent and independent pathways. Numerous studies have indicated that Bcl-2 family proteins play a crucial role in the regulation of apoptosis [26,27]. Consistent with the loss of the mitochondrial membrane potential, AG treatment also resulted in the modulation of mitochondrial proteins Bcl-2 and Bax. AG decreased the expression of the anti-apoptotic protein Bcl-2 while increasing the expression of the pro-apoptotic protein Bax (Figure 2C, Figure S2: Uncropped Western blot images), further supporting the participation of mitochondria in AG-induced apoptosis of FaDu cells.

At 24 h, treatment with 25 µM andrographolide (AG) markedly increased DNA damage in FaDu cells, as evidenced by a substantial increase in the average comet tail length compared with the untreated control (*p* < 0.0001). In contrast, 10 mM NAC alone did not significantly alter comet tail length compared with the control. Notably, co-treatment with NAC + AG markedly reduced the AG-induced increase in comet tail length, which was comparable with the control (Figure 2D). These findings suggest that AG induces substantial DNA damage in FaDu cells, while NAC effectively attenuates AG-induced DNA damage, supporting the involvement of oxidative stress in AG-mediated genotoxicity.

Following treatment with AG, Chk1 and Chk2, the cell cycle checkpoint kinases downstream of ATM/ATR, remained phosphorylated at the Ser345 and Thr68, respectively, at 24–48 h of treatment (Figure 2E, Figure S3: Uncropped Western blot images). ATM is generally activated in response to double-strand breaks (DSBs) in DNA damage [28,29] leading to phosphorylation of histone H2A.X, which initiates the assembly of specific DNA repair enzymes [30,31]. AG induced strong phosphorylation of H2A.X(S139) at 24 and 48 h of treatments, indicating DNA damage (Figure 2E).

### 3.3. AG Induces DNA Damage and Apoptosis in CAL-33 Cells

**Figure 3:**
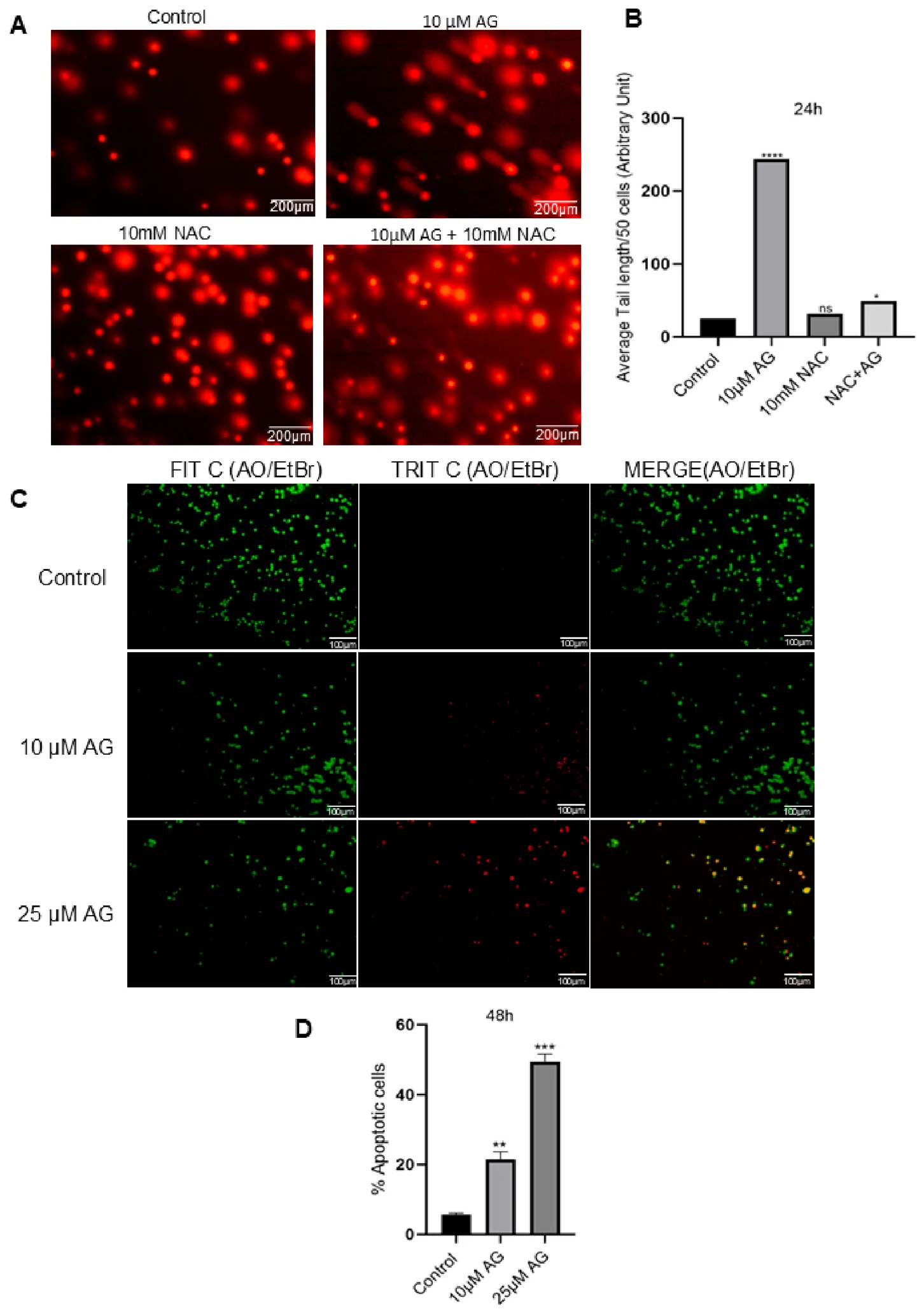
Effect of AG on DNA damage and apoptosis in CAL-33 cells. Cells were treated with either DMSO, 10µM AG and 10mM NAC for 24 h and 48 h as indicated in the figure. (**A**,**B**) Comet assay was done in CAL-33 cells at 24 h of AG treatment (25 µM), photographs were captured at 20X magnifications as described in Materials and Methods, and tail length measured; scale, 200 µm. (**C**,**D**) AO/EtBr was performed as described, and percent apoptotic cells was plotted. Bars, SE; * *p* < 0.05, ** *p* < 0.01, *** *p* < 0.001, **** *p* < 0.0001 when compared to the control.

To investigate the involvement of oxidative stress in andrographolide (AG)-induced DNA damage, an alkaline comet assay was performed in CAL-33 cells following treatment with 10µM AG alone, 10mM NAC alone, and the AG + NAC combination for 24 h. Compared with the untreated control group, AG-treated cells exhibited a pronounced increase in DNA migration, as evidenced by increased comet tail formation, indicating enhanced DNA damage (Figure 3A). In contrast, 10mM NAC treatment alone produced minimal or no changes in comet formation compared with the control group. Notably, treatment with NAC and AG treatment substantially reduced the DNA migration induced by AG, as reflected by a reduction in comet tail formation compared with the AG-alone group. These findings suggest that NAC partially attenuates AG-induced DNA damage in CAL-33 cells, indicating a potential contribution of oxidative stress to AG-mediated genomic damage (Figure 3A,B). We further confirmed apoptosis induction by acridine orange/ethidium bromide (AO/EtBr) staining (Figure 3C). Treatment with AG alone for 48 h led to an increase in apoptotic cells by 20% (*p* < 0.01) and 48% (*p* < 0.001) after 10 and 25 µM doses, respectively, in a dose-dependent manner, as compared to 5.3% in the control group of CAL-33 cells (Figure 3D).

### 3.4. Combination of AG and AZD7762 Shows Synergy with Regards to Inhibition of Cell Proliferation and Increase in Apoptosis

**Figure 4:**
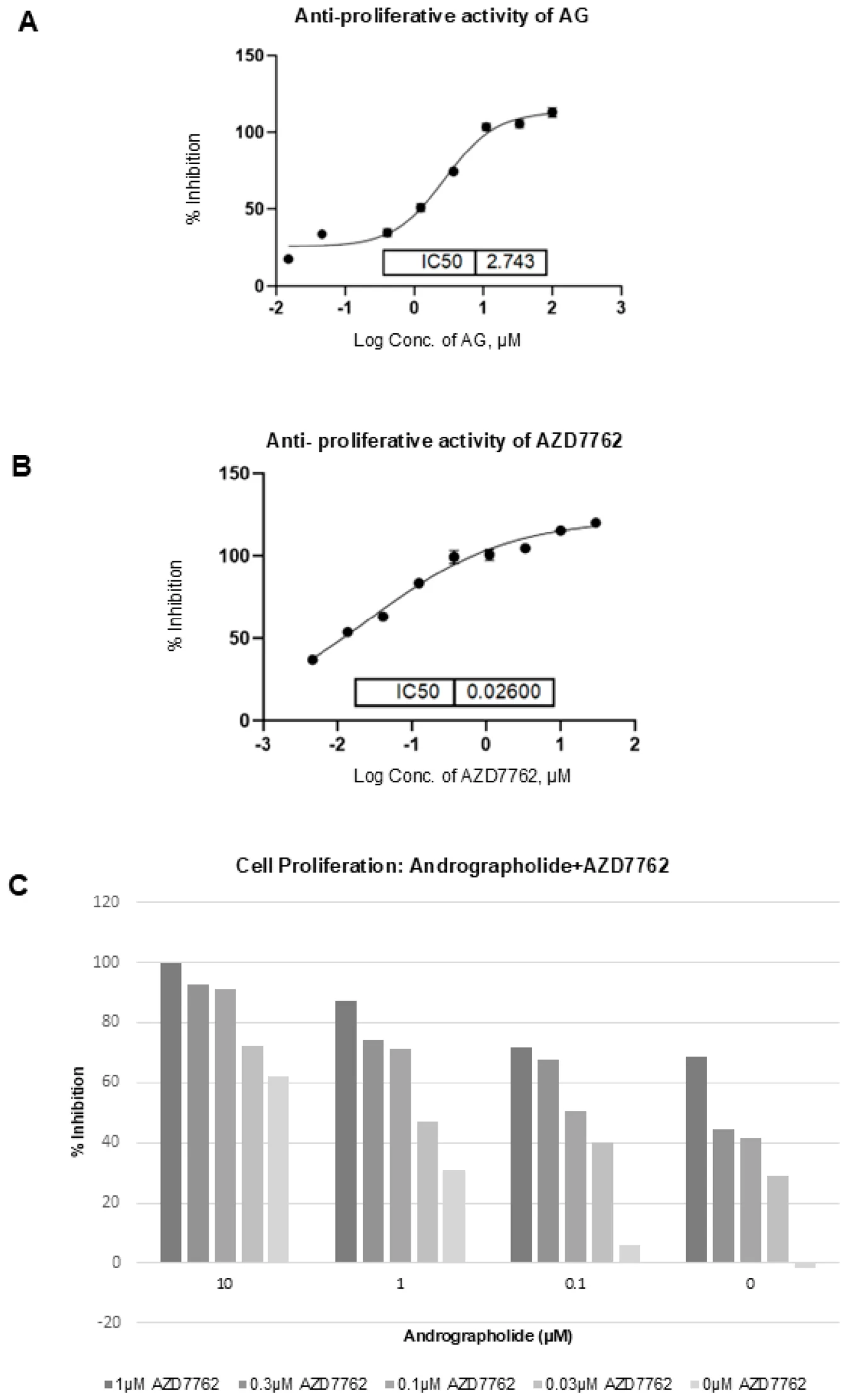
Cell proliferation studies in CAL-33 cells with AG and AZD7762 combination using MTT assay. CAL-33 were treated with AG and AZD7762 in a 3-fold serial dilution for 72 h. Cell viability was assessed by MTT assay. (**A**,**B**) Log-transformed AG and AZD7762 concentrations were plotted against percent inhibition to generate sigmoidal dose–response curves. Nonlinear regression analysis using a four-parameter logistic model (GraphPad Prism) was performed to determine IC_50_. (**C**) CAL-33 cells were exposed to varying concentrations of AG and AZD7762 combinations for 72 h, cell viability was measured using the MTT assay, and percent inhibition was plotted against drug concentration. Doxorubicin was included as a positive control to confirm assay sensitivity. Data is representative of three independent experiments showing a similar dose-dependent reduction in cell viability following combination treatment.

To assess the anti-proliferative effect, CAL-33 cells were treated with different drug concentrations of AG and AZD7762 and the MTT assay was performed. Percent growth inhibition was calculated with respect to the vehicle control (DMSO) and positive control (10 μM Doxorubicin). The IC_50_ values for AG and AZD7762 were 2.743 μM and 0.02600 μM, respectively (Figure 4A,B). To further evaluate the additive and synergistic effects, AG and AZD7762 combinations were studied at multiple concentrations in a cell proliferation assay. The % inhibition obtained at 1 μM and 10 μM AG alone was 31.05% and 62.40%, respectively, whereas when 1 μM and 10μM AG were used in combination with 0.1μM AZD7762, the % inhibition increased to 70.99% and 91.04%, respectively. Similarly, when 1 μM and 10 μM AG were used in combination with 0.3 μM AZD7762, the % inhibition increased to 74.16% and 92.85%, respectively.

These studies clearly demonstrated that AG treatment in combination with AZD7762 in CAL-33 cells decreased cell viability in a dose-dependent manner and at much lower doses (Figure 4C). The combination effect was assessed by the Chou–Talaya method, which showed a combination index (CI) value of 0.4, indicating strong synergy. This suggests that the simultaneous induction of DNA damage by AG and inhibition of checkpoint signaling by Chk1/2 inhibition collectively enhance cytotoxicity beyond the expected additive effect. The strong synergism is indicative of a mechanistic interaction wherein checkpoint inhibition prevents DNA repair recovery and promotes apoptotic cell death. Therefore, we next evaluated the impact of combination treatment on the cell cycle. Based on this, we selected 1 μM and 10 μM AG and 0.1 μM and 0.3 μM AZD7762 for further combination studies.

### 3.5. AG–AZD7762 Combination Enhances DNA Damage and Impairs DNA Repair in CAL-33 Cells

Cell cycle distribution was analyzed by propidium iodide (PI) staining following 24 h of treatment. Single-agent treatments of 1 μM AG and 10 μM AG resulted in G2/M populations of 28.2% and 47.6%, respectively, as compared to 21.9% in the untreated control, which was comparable to our previous studies [32]. Similarly, single-agent treatments of 0.1 μM AZD7762 and 0.3 μM AZD7762 resulted in G2/M populations of 31.1% and 56.7%, respectively.

**Figure 5:**
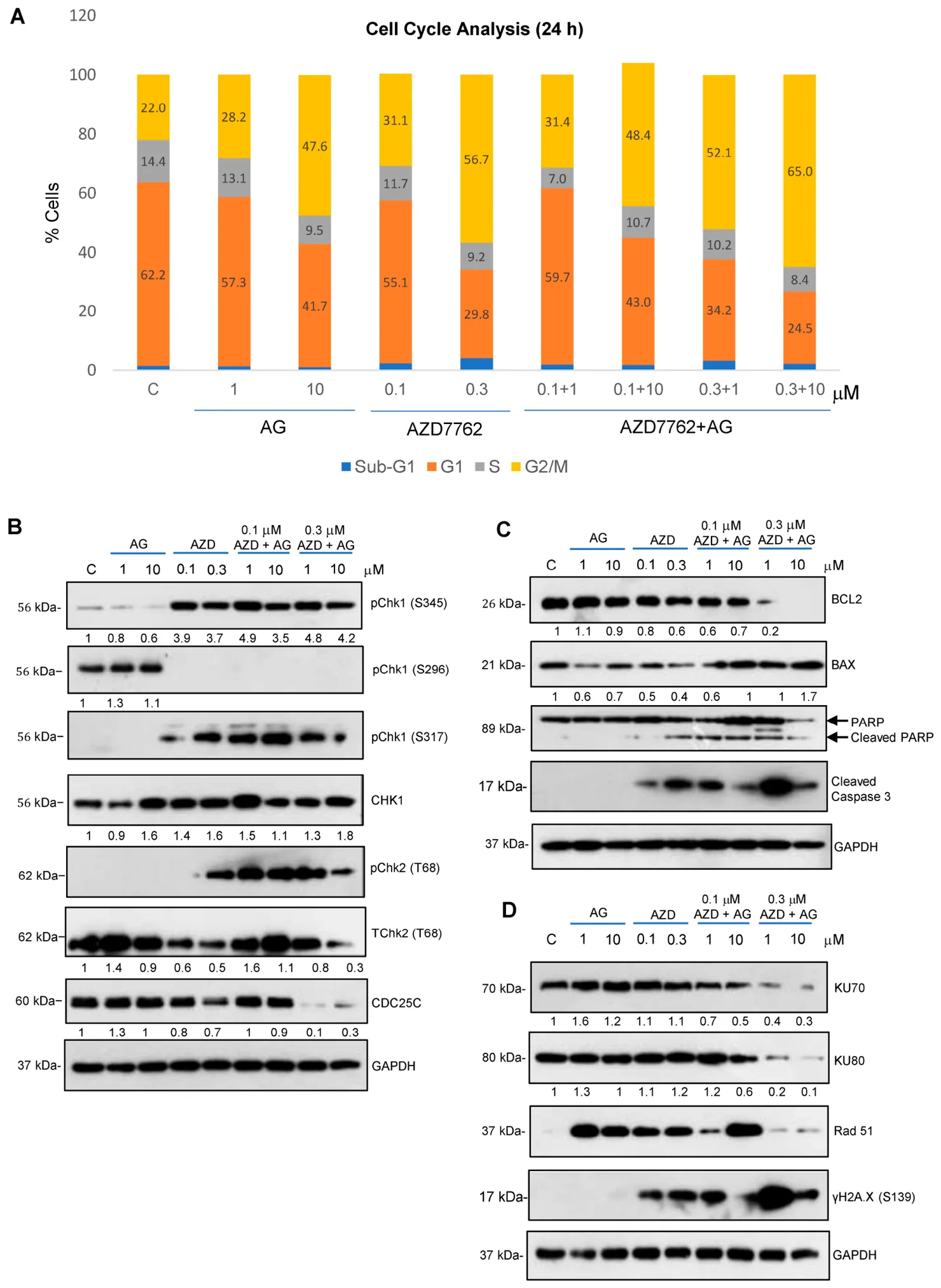
Cell cycle modulation and apoptosis induction by combination of AG and AZD7762 in CAL-33 cells. (**A**) Effect of AG and AZD7762 on cell cycle distribution. CAL-33 cells were treated with AG and AZD7762 for 24 h, followed by propidium iodide (PI) staining to evaluate cell cycle distribution by flow cytometry. Quantitative analysis revealed a significant increase in the G2/M population at 24 h time point when treated in combination compared to vehicle control. (**B**) Western immunoblot of ATM/ATR and Chk 1/2 signaling-associated proteins, CDC25C and (**C**) Western immunoblot of proteins involved in apoptosis. (**D**) Western immunoblot of KU70, KU80, Rad51, and γ-H2AX, as described in Materials and Methods. Numbers below the bands represent fold changes from the control as determined by densitometric analysis normalized to GAPDH using ImageJ 1.54g software.

The combination treatment of AG and AZD7762 resulted in a further increase in the G2/M population compared to single-agent treatments in a dose-dependent manner. The combination of 10 μM AG with 0.1 and 0.3 μM AZD7762 resulted in G2/M populations of 48.4% and 65%, respectively (Figure 5A) Similarly, 1 μM AG in combination with 0.1 and 0.3 μM AZD7762 resulted in G2M populations of 31.4% and 52.1%, respectively (Figure 5A). Overall, combinations exhibited stronger G2/M cell cycle arrest compared to the Chk1 inhibitor or AG alone, suggesting enhanced DNA damage induced by the combination.

Chk1/2 are key mediators of the DNA damage response and were found to be activated following AG treatment (Figure 2E), likely as a cellular response to AG-induced DNA damage. Based on this observation, we hypothesized that pharmacological inhibition of Chk1/2 would impair DNA damage signaling and repair, thereby enhancing AG-induced DNA damage and apoptotic cell death even at lower concentrations. Accordingly, AG was evaluated in combination with AZD7762.

Phosphorylation at Ser296 is a well-established marker of Chk1 activation, and subsequent Chk1/2-mediated signaling [33]. Accordingly, treatment with AZD7762 alone and in combination doses resulted in a marked reduction in Chk1 autophosphorylation at Ser296, indicating effective target engagement and inhibition of Chk1 kinase activity (Figure 5B, Figures S4 and S5: Uncropped Western blot images).

Chk1 (Ser345, and Ser317), on the other hand, are primarily phosphorylated by upstream ATM/ATR kinases in response to DNA damage and replication stress [34]. Accordingly, phosphorylation of Chk1 at Ser345 and Ser317 were significantly up-regulated by all the treatments (Figure 5B) and did not show any significant changes with Chk1/2 inhibitor treatment, suggesting that Chk1/2 functions downstream of ATM/ATR and modulation of DNA damage response by a Chk1/2 inhibitor is probably independent of ATM/ATR signaling.

To study whether accumulated DNA damage triggered apoptotic cell death, expression of key apoptosis regulators was determined. Our current studies and previous findings demonstrate that AG modulates the expression of BAX and BCL2 to cause apoptosis via the intrinsic mitochondrial pathway [35]. Consistent with this, protein-level analysis showed an increase in the expression of pro-apoptotic protein BAX along with downregulated expression of anti-apoptotic protein BCL-2 in a dose-dependent manner (Figure 5C, Figure S6: Uncropped Western blot images) [36,37]. This shift toward a pro-apoptotic state suggested that cells experiencing unrepaired DNA damage are directed toward programmed cell death.

Further validation of apoptosis induction was done through analysis of cleaved PARP and cleaved Caspase 3. Treatment with the combination resulted in a significant increase in the expression of cleaved PARP and cleaved Caspase 3 compared with control and single agents alone, indicating activation of caspase-dependent apoptotic pathways. These results suggest that checkpoint inhibition and DNA damage accumulation ultimately culminate in apoptotic cell death [38].

γH2AX (H2AX phosphorylation) is an established marker of DNA damage that is regulated by the ATM and ATR kinases. It initiates downstream pathways that control apoptosis and cell cycle checkpoints, and decides the cellular fate between cell death and repair [39]. To find out whether checkpoint abrogation leads to increased genomic instability, γH2AX expression was assessed by Western blotting. Stronger increase in γH2AX levels was found in the combination treatments compared with the controls, AG alone and AZD7762 alone (Figure 5D, Figure S7: Uncropped Western blot images), indicating enhanced accumulation of DNA damage, likely resulting from the loss of checkpoint-mediated DNA repair [40]. To confirm this, we assessed DNA repair machinery following combination treatment. Given the marked accumulation of DNA damage following combination treatment, we next assessed the expression of key proteins involved in homologous recombination (HR) and non-homologous end joining (NHEJ) DNA repair pathways. A marked decrease in RAD51 expression was observed following treatment with the combination doses (0.3 μM AZD7762 + 1 μM AG and 0.3 μM AZD7762 +10 μM AG), suggesting an impaired homologous recombination (HR) repair capacity and a reduced ability to repair DNA double-strand breaks. Downregulation of RAD51 (Figure 5D) further supports disruption of HR-mediated DNA repair mechanisms [41].

Western blot analysis revealed a significant decrease in the expression of KU70 and KU80, key components of the non-homologous end joining (NHEJ) pathway (47), following treatment with the combination (0.3μM AZD7762 with 1μM and 10μM AG) (Figure 5D). The KU70/KU80 heterodimer is responsible for recognizing DNA double-strand breaks and recruiting DNA-PK to initiate NHEJ repair. The coordinated downregulation of KU70 and KU80 suggests impaired NHEJ repair activity, thereby reducing the ability of cells to repair DNA double-strand breaks through this pathway [42].

Overall, the simultaneous suppression of both HR and NHEJ repair pathways indicates a profound impairment of DNA repair capacity, which likely contributes to the extensive accumulation of DNA damage observed following combination treatment.

## 4. Discussion

HNSCC is one of the cancers for which limited treatment options are available that offer marginal therapeutic benefits and are often associated with acute and long-term treatment-associated complications [43]. Therefore, an effective and safe treatment option is still a high unmet need for HNSCC. One of the central findings in this study is that andrographolide (AG) exerted its anticancer effects through inhibition of cell proliferation and cell cycle progression, and induction of DNA damage and oxidative stress leading to apoptosis in HNSCC cells. It was noteworthy that the anti-proliferative and pro-apoptotic effects of AG on HNSCC were not observed in normal human intestinal epithelial FHs74int cells. Also, a previous study by Yang et al. [44] states that andrographolide showed little to no cytotoxicity in normal human oral keratinocytes (NHOKs) at the concentrations that strongly suppressed oral cancer stem-like cells. These results and a few other earlier publications [45,46,47] suggest that AG has the potential to be a selective anticancer agent against cancer cells, sparing normal cells, although additional animal studies are needed to confirm the same.

Inhibiting deregulated cell cycle progression is an effective strategy to suppress cancer growth, with the G2/M checkpoint playing a pivotal role in preventing cells harboring damaged DNA from entering mitosis [48]. In our previous study, AG induced a significant accumulation of CAL-33 cells in the G2/M phase, indicating apoptosis and activation of the DNA damage-induced checkpoint [32]. Apoptosis in CAL-33 cells was further confirmed by AO/EtBr staining and by assessing the levels of cleaved Caspase and PARP. Mechanistically, DNA damage activates the ATM/ATR kinases, which phosphorylate Chk2 and Chk1, respectively, resulting in the suppression of Cdc25 phosphatases and the inactivation of the Cyclin B1–CDK1 complex, thereby ensuring G2/M arrest and permitting time for DNA repair [49]. Considering the multiple resistance mechanisms and complex oncogenic signaling pathways in malignancies, monotherapy may prove to be rather ineffective for cancer patients. This may explain why combination therapies are often the way for cancer treatment and why, in the era of precision medicine, combination drug therapies are a more promising strategy [50]. Although AG demonstrating selective cytotoxicity towards CAL-33 cells at higher concentrations such as 25μM with negligible damage to normal cells was an advantage, its anticancer effectiveness as a monotherapy was constrained at lower concentrations.

In this case, DNA repair mediated by Chk1/2 pathways appears to be a biologically relevant combination to enhance the potency of AG. Since the Chk1/Chk2 signaling system mediates DNA repair, which is a biologically significant mechanism that contributes to cancer cell survival, blocking this pathway offers as a good strategy to increase the anticancer efficacy of AG. Therefore, we explored the combination of AG with the Chk1/2 inhibitor AZD7762 to check if checkpoint abrogation and defective DNA repair could sensitize CAL-33 cells to AG-induced DNA damage, hence enhancing therapeutic efficacy at lower, relevant concentrations. In particular, compared to AG alone, which required about 25 μM to produce a similar apoptotic response, the combination of AG and the Chk1/2 inhibitor AZD7762 caused noticeably more apoptosis at much lower AG doses (1–10 μM) in CAL-33 cells. These data indicate that Chk1/2 inhibition significantly potentiates the anticancer effectiveness of AG, permitting strong apoptosis at lower, possibly relevant concentrations in CAL-33 cells. Chk1/2 pathways are clinically validated and many inhibitors are in clinical trials that stand alone or are used in combination with other approved chemotherapy agents. Additionally, a phase I combination study of irinotecan and AZD7762 has also been conducted [51] suggesting the relevance of the Chk1/2 pathway, specifically in the context of potentiating DNA damage. Still, further in vitro and in vivo studies are required to better understand its mechanism of action and clinical utility in this specific context.

Nevertheless, in later stages of malignancies, cancer cells are known to become resistant to apoptosis and/or become insensitive to DNA damage induced by chemotherapeutic agents. AG sensitizes the cancer cells by tipping the balance towards pro-apoptotic signaling by increasing Bax and decreasing BCl-2 expression, further resulting in mitochondrial membrane depolarization, which leads to apoptosis [52]. In turn, it leads to activation of caspase-9 and 3, followed by cleavage of PARP. Therefore, AG triggered caspase-dependent apoptosis and acted as a potent chemotherapeutic agent against HNSCC cells. AG has been reported to induce cell death via apoptosis in colon cancer cell lines via activation of the ER stress-mediated GRP78/IRE1/XBP1/CHOP signaling cascade. Moreover, in MDA-MB-231 breast cancer cells, AG promotes apoptosis by increasing intracellular ROS production and disrupting the mitochondrial membrane potential [53].

It is known that DNA damage results in mutations or genomic instability, which may result in cancer initiation as well as progression; however, causing DNA damage, such as DNA double-strand breaks (DSBs), has been shown to be an effective mechanism to kill cancer cells [54]. We observed that AG-induced cell death involves DNA damage in CAL-33 cells as observed using the alkaline comet assay (Figure 3A,B). Although we observed that AG increases intracellular level of ROS, inducing DNA damage and causing cell death, which was reversed by pre-treatment of CAL-33 cells with NAC, the precise mechanism of ROS accumulation warrants further investigations.

Chk1 and Chk2, the cell cycle checkpoint kinases downstream of ATM/ATR, remained phosphorylated at the Ser317 and Thr68, respectively, at 24–48 h of treatment [40,55]. Since AG induces DNA damage, increased activation of CHK1/2 proteins suggest a natural defensive response by the cells. Once triggered, ATM phosphorylates downstream targets, including Chk1 and Chk2 and hence leads to cell cycle arrest. This pause in the cell cycle provides time for DNA repair and promotes cell survival [56]. The activation of these repair pathways involving Chk1/2 in response to AG treatment suggested that cancer cells are attempting to counteract the drug-induced DNA damage. Inhibiting Chk1/2 in this context would interfere with checkpoint control, preventing adequate DNA repair and forcing damaged cells to progress through the cell cycle. Also, in our previous studies we observed G2/M cell cycle arrest in CAL-33 cells when treated with 5, 10 and 25 μM AG [32]. In the present study, CAL-33 in combination with a Chk1/2 inhibitor (AZD7762) resulted in an increase in Chk1 phosphorylation at S345. Upon DNA damage, ATR activates Chk1 at two established sites, S345 and S317, leading to autophosphorylation at S296. Notably, previous studies have demonstrated that pS345 Chk1 (phosphorylated S345 Chk1) is increased in response to Chk1 inhibition [33].

Under these conditions, CAL-33 cells were unable to recover from AG-induced damage in the presence of AZD7762 and were rather pushed to undergo apoptotic cell death. Previous studies have shown that combining DNA-damaging agents with Chk inhibitors enhances cytotoxicity by overwhelming the cellular repair machinery [36]. In our study, inhibition of Chk1/2 checkpoints along with andrographolide lead to inhibition of NHEJ repair machinery as seen by decreased expression of Rad51, KU70, and KU80 [33,55]. The notable increase in the G2/M population in CAL-33 following combination therapy in dose-dependent manner suggests premature mitotic entry of these cells leading to increased apoptotic cell death, which is strongly supported by coordinated changes in DNA damage checkpoint and apoptotic signaling proteins. The increase in phosphorylated Chk1 at Ser317 and Ser345 in combination treatment implies inhibition of ATR-Chk1-mediated checkpoint activation, a mechanism necessary for the replication stress response and DNA repair [57,58]. A similar increase in phosphorylation of Chk2 at T68, a known substrate for ATM kinase, further indicates enhanced DNA damage with the AG/Chk1/2 inhibitor combination.

Consistently, Chk1 inhibition is known to abrogate S and G2/M checkpoints thereby promoting cell cycle progression despite the presence of DNA damage [56]. The observed decrease in CDC25C further indicates disruption of proper G2/M regulatory control, as CDC25C is a key downstream effector of CHK1 signaling [59]. Overall, our results suggest that an AG and Chk1/2 inhibitor combination treatment results abrogates G2 cell cycle arrest, and results in premature mitotic entry of cells with DNA damage, thereby leading to profound apoptotic cell death.

Apoptotic induction was further confirmed by increased BAX expression, decreased BCL2 levels, elevated cleaved caspase-3, and increased cleaved PARP. The rise in cleaved caspase-3 and cleaved PARP along with inhibition on PARP in the combination treatment establishes activation of the intrinsic caspase-dependent apoptotic pathway [60,61]. An increased BAX/BCL2 ratio facilitates mitochondrial outer membrane permeabilization, resulting in the release of cytochrome *c* and activation of downstream caspases that drive apoptosis. Collectively, these findings indicate that andrographolide resulted in abrogation of the G2 checkpoint and DNA damage-mediated apoptosis at much lower doses when combined with AZD7762 as compared to its effect as a single agent, clearly suggesting that the Chk1/2 pathway could be targeted to potentiate the DNA damage response of chemotherapeutic agents.

## 5. Conclusions

Our studies and literature clearly show the potential of AG as an anticancer agent. However, its moderate potency and poor oral bioavailability make it imperative to identify combination drugs to be used with AG to make it an effective anticancer agent. The Chk1/2-mediated DNA damage response was activated by treatment with AG, which resulted in effective DNA repair and impaired apoptosis, and therefore inhibition of the DNA damage response enhanced AG mediated anticancer activity. Accordingly, the combination of Chk1/2 inhibitor AZD7762 with AG resulted in a stronger apoptosis of CAL-33 cells at much lower doses. However, further investigation of the AG/AZD7762 combination in FaDu and other HNSCC cells is warranted to establish the broader applicability and generalizability of these findings. Such mechanism-based combinations are a promising approach for cancer therapy and warrant further mechanistic and pharmacological studies.

## Data Availability

The original contributions presented in this study are included in the article/Supplementary Materials. Further inquiries can be directed to the corresponding author.

## Supplementary Materials

The following supporting information can be downloaded at: https://www.mdpi.com/article/10.3390/cancers18193199/s1, Figure S1: Cell cycle distribution when treated with AZD7762 and andrographolide in 1 AG, 10 AG, 0.1 AZD7762, 0.3 AZD7762, 1 AG + 0.1 AZD7762, 10 AG + 0.1 AZD7762, 1 AG + 0.3 AZD7762, and 10 AG + 0.3 AZD7762 μM concentration for 24 h time period. The combination treatment of AG and AZD7762 resulted in a marked increase in the G2/M population in CAL-33 compared to control and single-agent treatments in a dose-dependent manner (Figure 5A); Figure S2: Original images of Western blots for Figure 2C; Figure S3: Original images of Western blots for Figure 2E; Figure S4: Original images of Western blots for Figure 5B. Left panel; Figure S5: Original images of Western blots for Figure 5B. Left panel; Figure S6: Original images of Western blots for Figure 5C. Right panel; Figure S7: Original images of Western blots for Figure 5D.
