# Supplementary material for: Chk1/2 Inhibition by AZD7762 Enhances Andrographolide-Induced DNA Damage, Impairs DNA Repair, and Promotes Apoptosis in Head and Neck Squamous Cell Carcinoma CAL-33 Cells
Source: Cancers (Basel). 2026 Oct 3;18(19):3199. doi: 10.3390/cancers18193199 (PMC13664140; doi:10.3390/cancers18193199)
Supplement: Supplementary file 1 [file cancers-18-03199-s001.zip › cancers-4467503-supplementary.pdf]

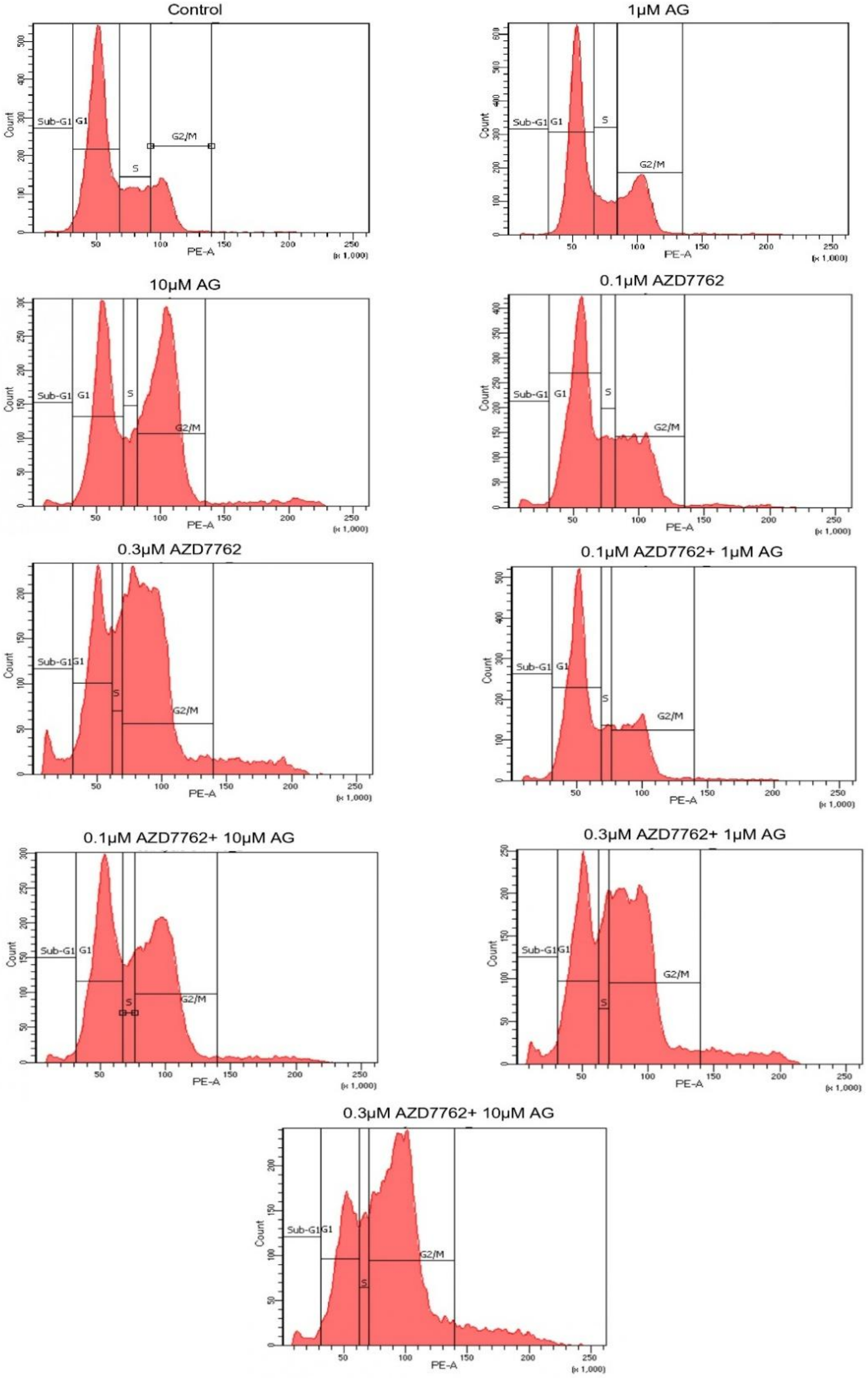

Figure S1: Cell cycle distribution when treated with AZD7762 and andrographolide in 1AG, 10AG, 0.1AZD7762, 0.3 AZD7762, 1AG +0.1 AZD7762, 10AG+0.1 AZD7762, 1AG+0.3 AZD7762, 10AG +0.3 AZD7762 µM concentration for 24hr time period. The combination treatment of AG and AZD7762 resulted in a marked increase in the G2/M population compared to control and single-agent treatments in a dose dependent manner (Figure.5A)

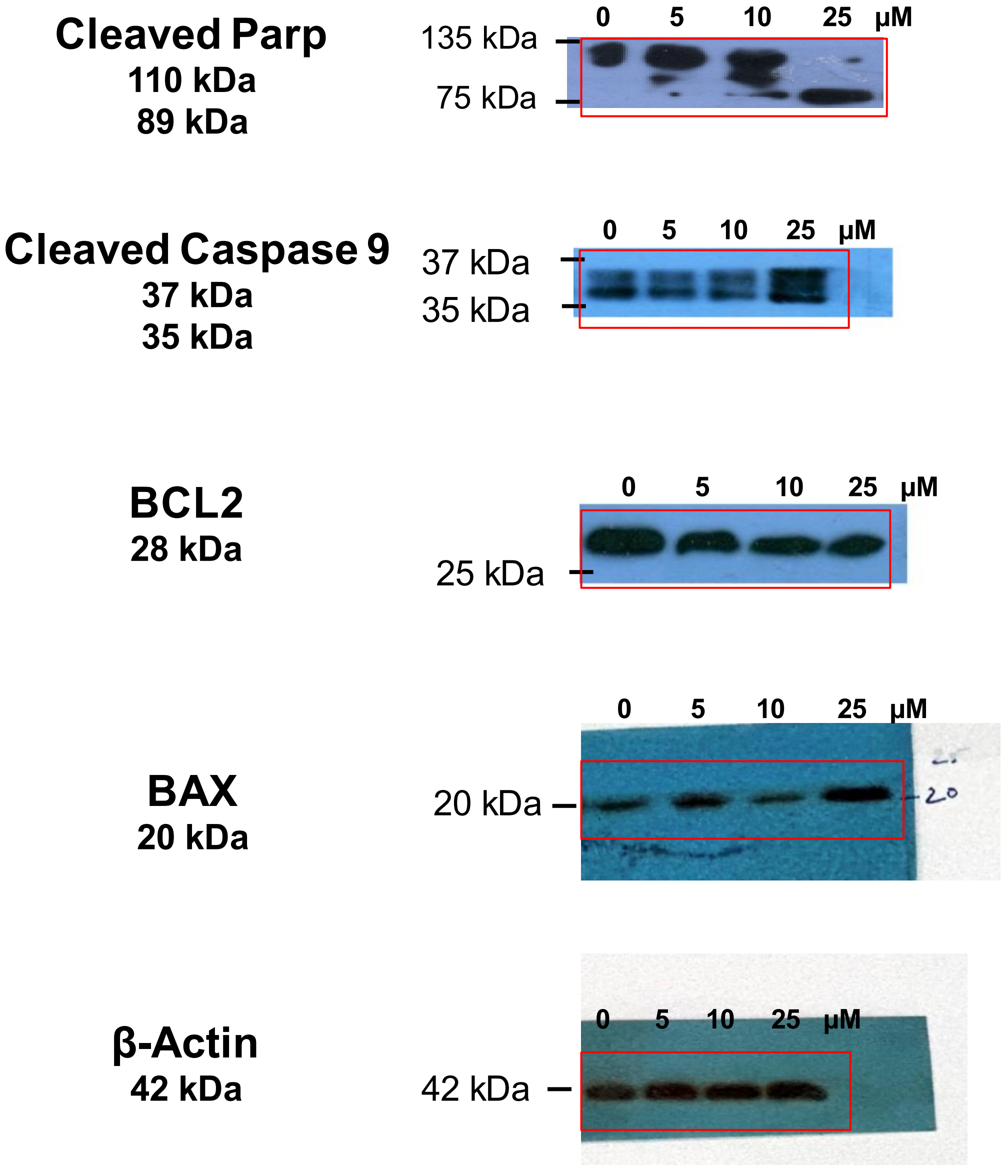

Figure S2. Original images of western blots for Figure 2C.

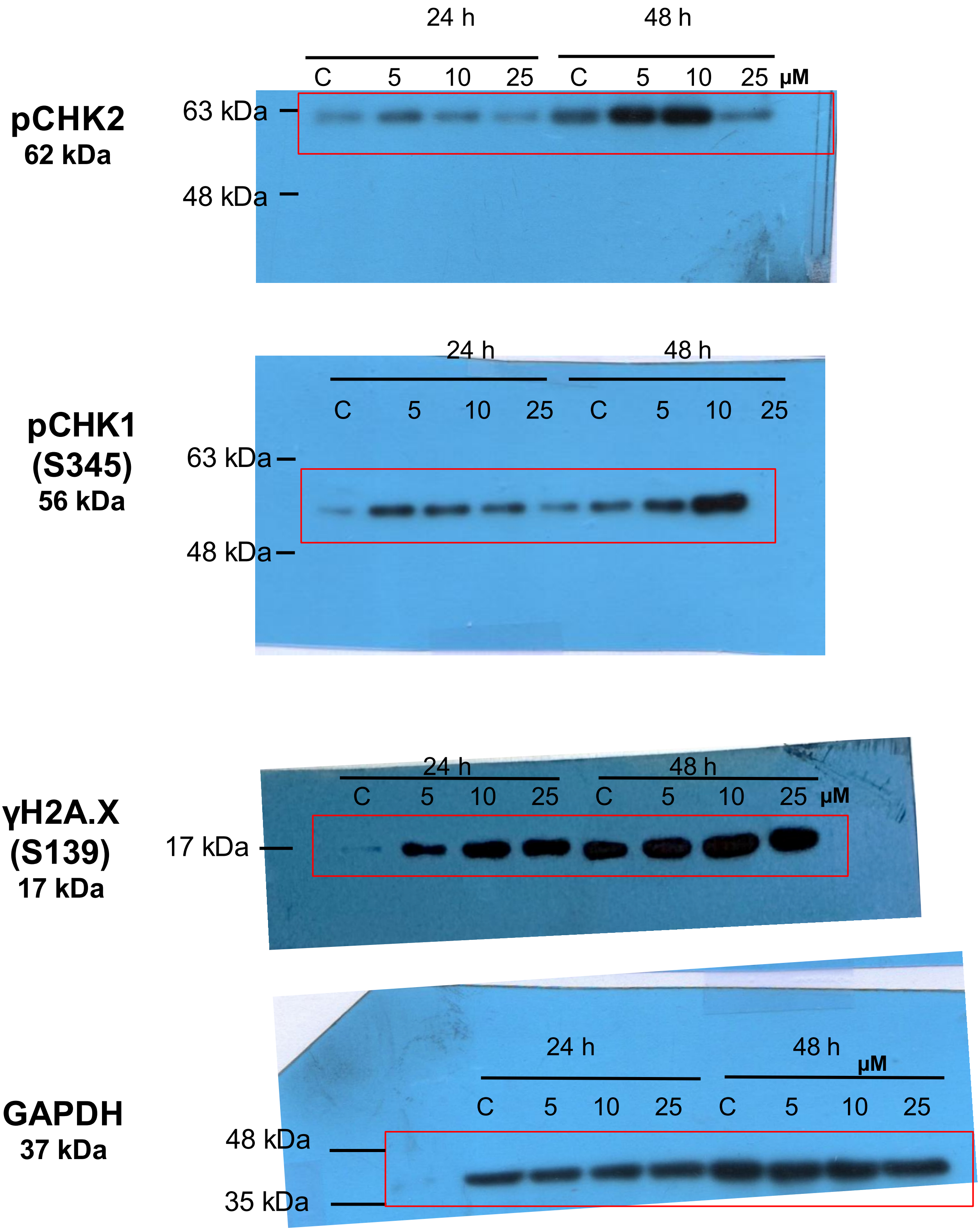

Figure S3. Original images of western blots for Figure 2E.

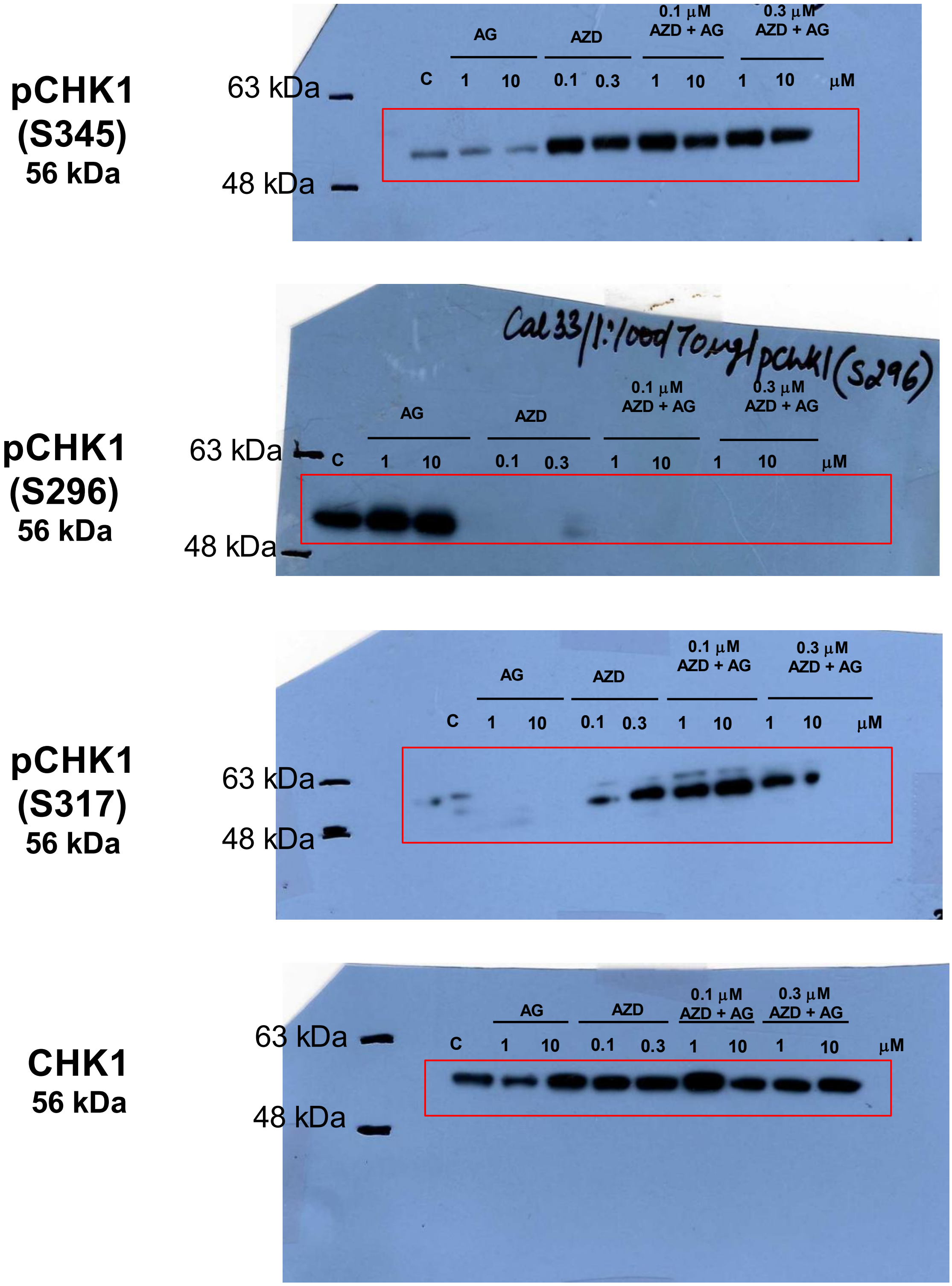

Figure S4. Original images of western blots for Figure 5B. left panel.

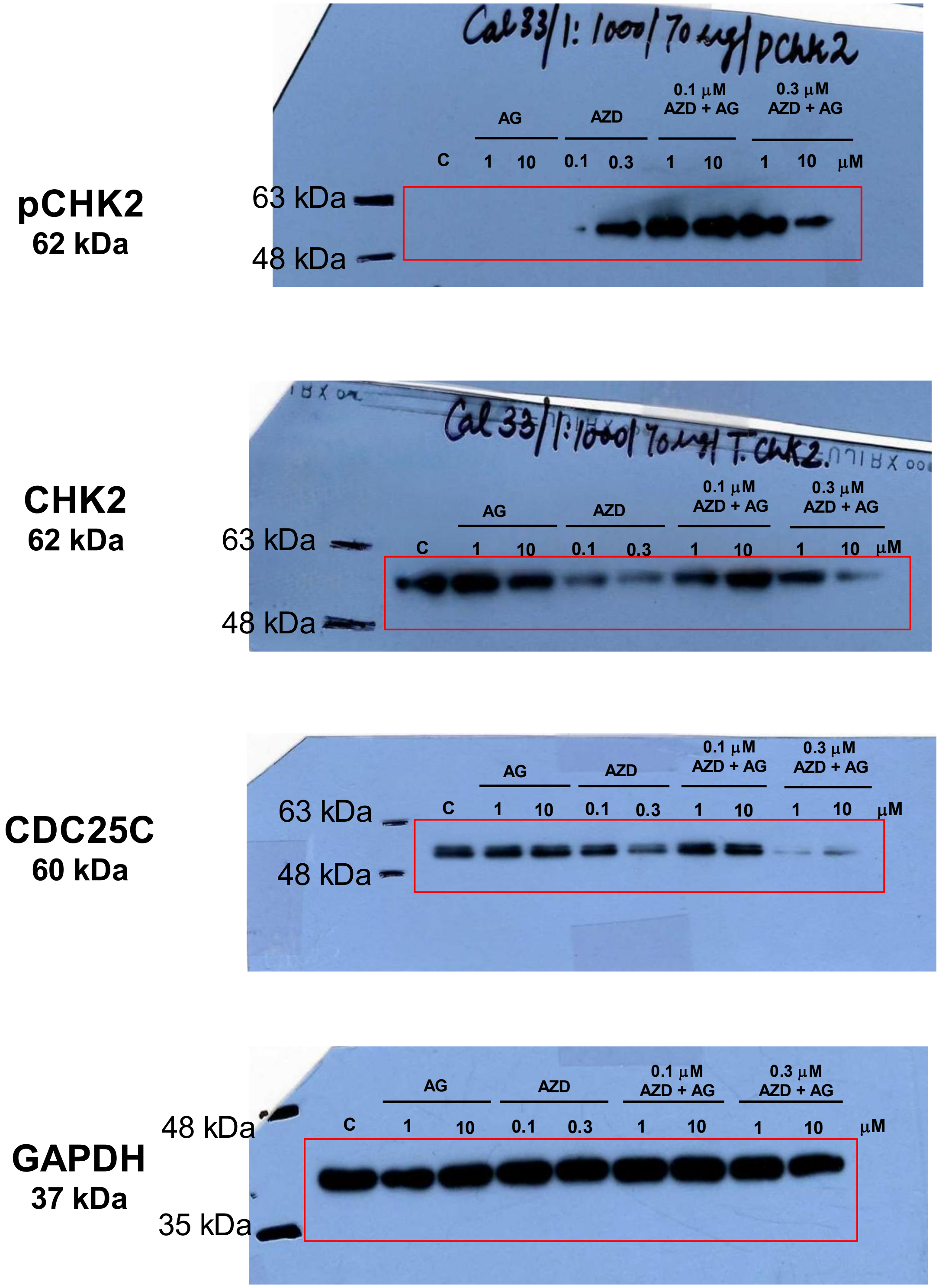

Figure S5. Original images of western blots for Figure 5B. left panel.

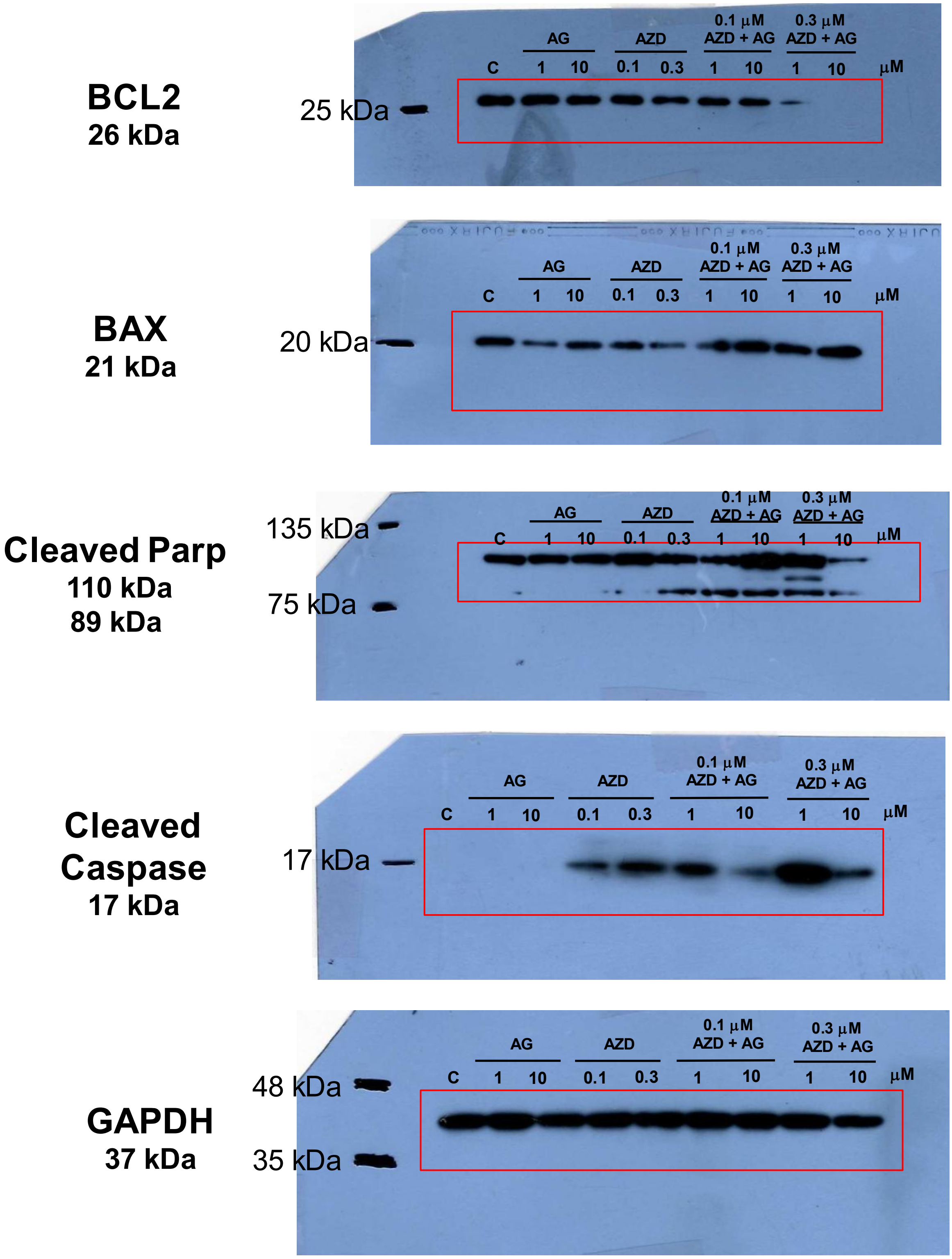

Figure S6. Original images of western blots for Figure 5C. Right panel.

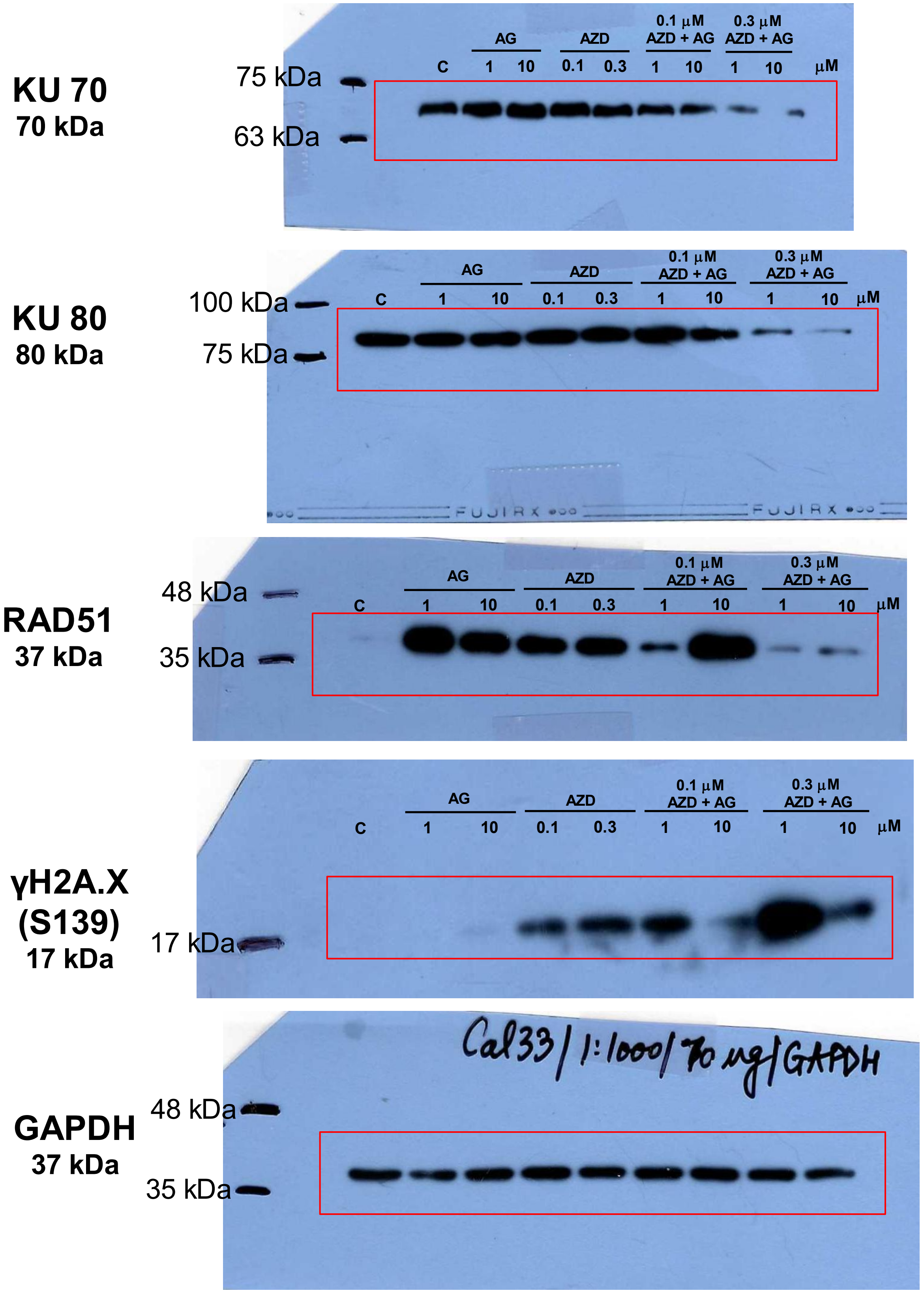

Figure S7. Original images of western blots for Figure 5D.
